# Supplementary material for: A Global Screen for Assembly State Changes of the Mitotic Proteome by SEC-SWATH-MS
Source: Cell Syst. 2020 Feb 26;10(2):133–155.e6. doi: 10.1016/j.cels.2020.01.001 (PMC7042714; doi:10.1016/j.cels.2020.01.001)

Q96DB5 | RMD1_HUMAN | RMDN1 FAM82B CGI−90
Monomer MW [kDa]: 35.808 Monomer expected elution fraction: 49

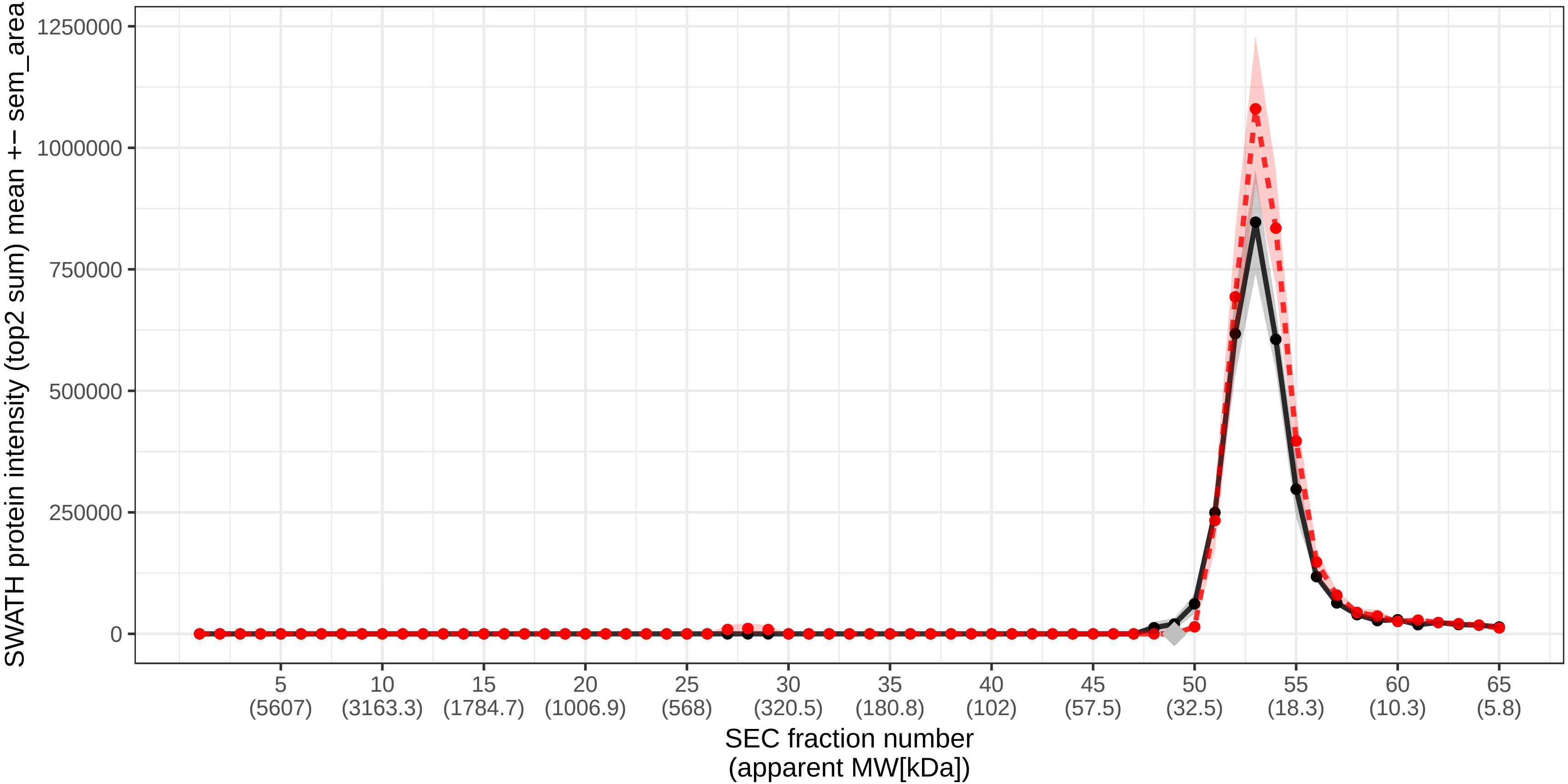

Supplement: Data S1. SEC-SWATH-MS Protein Chromatograms, Related to Figure 1 [file mmc6.zip › SECchrom_Q96DB5_RMD1_HUMAN_RMDN1_FAM82B_CGI-90.pdf]
